# Sputum-Based Molecular Biomarkers for the Early Detection of Lung Cancer: Limitations and Promise

**DOI:** 10.3390/cancers3032975

**Published:** 2011-07-19

**Authors:** Connie E. Kim, Kam-Meng Tchou-Wong, William N. Rom

**Affiliations:** 1 Department of Medicine, Division of Pulmonary, Critical Care and Sleep Medicine. 462 First Avenue, NBV 7N24, New York, NY 10016, USA; E-Mails: connie.kim@nyumc.org (C.E.K.); kam-meng.tchou-wong@nyumc.org (K.-M.T.-W.); 2 Department of Environmental Medicine, New York University School of Medicine, 57 Old Forge Road, Tuxedo, NY 10987, USA

**Keywords:** lung cancer, non-small cell lung cancer, sputum, biomarker, early detection

## Abstract

Lung cancer is the leading cause of cancer deaths, with an overall survival of 15% at five years. Biomarkers that can sensitively and specifically detect lung cancer at early stage are crucial for improving this poor survival rate. Sputum has been the target for the discovery of non-invasive biomarkers for lung cancer because it contains airway epithelial cells, and molecular alterations identified in sputum are most likely to reflect tumor-associated changes or field cancerization caused by smoking in the lung. Sputum-based molecular biomarkers include morphology, allelic imbalance, promoter hypermethylation, gene mutations and, recently, differential miRNA expression. To improve the sensitivity and reproducibility of sputum-based biomarkers, we recommend standardization of processing protocols, bronchial epithelial cell enrichment, and identification of field cancerization biomarkers.

## 1. Introduction

Lung cancer is the leading cause of cancer deaths in both men and women in the United States, with an overall 5-year survival rate of 10–15% that has persisted for decades [1]. There are two types of lung cancer: small cell lung cancer (SCLC) and non-small cell lung cancer (NSCLC). About 85% of all lung cancers are NSCLC, which can be further divided into three main types: large cell carcinoma; squamous cell carcinoma; and adenocarcinoma of the lung. Adenocarcinoma of the lung is the single most common type of lung cancer; it accounts for about 40% of all lung cancers. Although NSCLC grows more slowly and is less aggressive than SCLC, the most efficient treatment for NSCLC is considered to be surgical resection of the tumor, while SCLC is remarkably sensitive to chemotherapy and radiation treatments. The low survival rate of NSCLC patients is related to the late presentation of patients with an unresectable tumor due to the lack of a validated screening approach for early detection. By early detection of precancerous microscopic lesion, patients can avoid developing clinically diagnosable cancer and high-risk healthy individuals can be prevented from developing cancer by timely chemoprevention. Therefore, early detection of lung cancer, followed by appropriate treatment, will significantly increase the survival rate.

Low-dose computerized tomography (CT) scan has suggested a promising possibility to detect lung cancer earlier. Compared to chest X-rays, CT screening significantly reduced mortality in a high-risk population by 20.3% based on the recently reported National Lung Screening Trial (NLST), a large randomized trial funded by the National Cancer Institute (NCI)[2]. Although it is promising, the limited impact of CT screening on mortality may be because it cannot distinguish non-calcified nodules (NCNs) that progress to cancer from the benign ones. It also has limited ability to differentiate between benign and malignant lesions of tumors, especially the centrally located ones [3], demanding a complementary screening tool to increase specificity of detection for cancer. False-positive CT screening results will lead to unnecessary invasive procedures, which can cause stress and economic burden. Therefore, there is still a demand for non-invasive, sensitive biomarkers in order to accurately identify the high-risk population for optimal treatments and minimize unnecessary invasive procedures caused by false-positive CT screening results. These biomarkers will be also useful to increase sensitivity for the detection of centrally located lung cancer and to determine the strategy for the therapeutic management of clinically uncertain nodules that are detected by CT screen.

Tobacco smoking is the single, most important risk factor for lung cancer. An estimated 90% of lung cancer is due to cigarette smoking, which also causes at least 18 other types of cancer [4]. It is well accepted that the repeated exposure of the respiratory tracts of smokers to tobacco-related carcinogens causes molecular genetic and epigenetic abnormalities that culminate in lung cancer. Consequently, smoking may create a field of molecular injury throughout the airway epithelium exposed to cigarette smoke and increase the susceptibility of an entire field or area to carcinogenesis (field carcinogenesis) [5]. This suggests that the smoke-induced molecular abnormalities found in the respiratory tract (regardless of its exact location) may mirror the progression of cancer and therefore can be used as potential biomarkers to detect lung tumor at an early stage. Thus, sputum that contains exfoliated airway epithelial cells is an ideal specimen type for the discovery of biomarkers for lung cancer.

Molecular analysis of sputum has been an active area for the investigation of lung cancer biomarkers for several reasons. Sputum is the most easily accessible body fluid that contains the pathogenically relevant cell type, namely, bronchial epithelial cells. These cells are typically exfoliated from the central airways, where the CT scan seems to have a blind spot when the cancer arises from here. This also suggests that it is possible to identify tumor cells in sputum. Additionally, the other airway epithelial cells found in sputum may also harbor important molecular information, supported by the field cancerization theory [5], which reflects the local milieu of the lung. High-risk smokers typically produce increased amount of sputum, which makes it feasible to obtain enough material for analysis. Finally, collecting sputum is non-invasive, fast, and economical, which are important characteristics to be an ideal specimen type for large-scale population screening.

In this review, we discuss various sputum-based molecular biomarkers and how each one has developed during the last decade. We then explore the strengths and weaknesses of each marker and discuss ways to overcome the limitations.

## 2. Sputum-Based Molecular Biomarkers

### 2.1. Cytology

Sputum cytology is the oldest technique that utilizes sputum in detecting lung diseases. Identified and developed by Saccomannao [6], sputum cytology was shown to detect premalignant changes in high-risk groups several years before a clinical diagnosis of lung cancer. This finding is supported by the concept that changes in the cytology of bronchial epithelial cells reflect the progress from inflammation to cancer [7]. However, it is not considered to be sensitive enough mainly because it requires skills that can identify subtle nuclear changes in cells that often comprise less than 5% of the sputum samples. The “subjectivity” of the pathologist performing the cytopathological classification raises concerns about its reliability and reproducibility due to the lack of standardization. The results of two large, randomized clinical trials support this notion [8,9]. In these studies, they compared annual chest X-ray (CXR) plus sputum cytology with CXR only. After nine years of follow-up, they noticed the mortality due to lung cancer was slightly lower in the double-screened group. However, it is arguable whether it could have been occurred by chance.

To overcome this limitation of low sensitivity and reliability, sputum cytology has often been used as a supplement to other screening methods. Lam *et al.* [10] reported that among the high-risk patients with sputum atypia (47%), who were followed by bronchoscopy and CT scans, 15% of these patients had lung cancer. Notably, approximately half of these patients had early-stage cancer. It was reported that the overall sensitivity of sputum cytology in combination with bronchoscopy and CT scan in detecting lung cancer was 71% for all histological types and 100% for squamous cell lung cancer located in the central airways. On the other hand, combining sputum atypia with FISH assay for the detection of gene copy number changes of specific cancer-related genes did not improve the diagnostic performance compared to FISH screening alone [11]. These inconsistent results demand further improvement in the application of sputum cytology for cancer screening. Automated image cytometry for objective and quantitative cytopathological imaging may greatly improve the sensitivity. An automated imaging system quantifying the DNA content had greater sensitivity (80%) than conventional cytology (4.2%) for the diagnosis of lung cancer [12]. Kemp *et al.* [13] performed multicenter validation trial to compare the automated sputum analysis using DNA cytometry to the conventional one and found that the automated method detected lung cancer with 75% sensitivity while conventional cytology only identified 16%. These studies suggest that an objective, automated imaging system in combination with adequate molecular biomarkers may be a promising tool for the screening of lung cancer.

### 2.2. Allelic Alteration

Microsatellites are stretches of DNA in which a short motif (usually one to five nucleotides long) is repeated ten to hundred times. Microsatellites are prone to mutations during replication due to the transient split of the two helical strands and slippage of the DNA polymerase complex at reannealing, which generates an insertion or deletion loop depending on the slippage direction [14]. Microsatellite instability (MSI) is a situation in which these gained or lost repeat units as a consequence of the mutation result in a somatic change in length. MSI is known to be associated with defective DNA mismatch repair machinery [15]. In hereditary non-polyposis colorectal carcinoma (HNPCC), in which MSI was first observed and is found frequently, MSI is caused by germline mutations in DNA mismatch-repair genes [16], suggesting the phenomenon of MSI is associated with tumorigenesis. Furthermore, changes in the microsatellite repeats are known to correlate with altered gene expression [17,18]. Thus, MSI in tumor is likely to lead to mutations and deregulated gene expression compared to normal tissue, which may be critical for understanding tumorigenesis.

Another form of allelic alteration that can be revealed by MSI analysis is the loss of heterozygosity (LOH). LOH represents the loss of one allele of a gene whereby the other allele is already inactivated. LOH is caused by a variety of genetic mechanisms, including physical deletion of chromosome nondisjunction, mitotic nondisjunction followed by republication of the remaining chromosomes, mitotic recombination, and gene conversion [19]. The frequent allelic loss at particular chromosomal regions in tumors is indicative of the presence of a tumor suppressor gene. Consequently, detection of LOH has been used to identify novel tumor suppressor genes. Furthermore, it is shown that some LOH patterns are associated with different tumor types, pathological stages, and progression [20,21].

Allelic imbalance in lung cancer has been actively investigated. Individual LOH or MSI is rarely used as a stand-alone biomarker because there are numerous of them existing through the human genome and the frequency of the occurrence of each marker hardly provides statistical significance. Therefore, the key is to find the right combination of MSI and LOH to increase the sensitivity and specificity. A few commonly chosen regions for assessing MSI and LOH include, but are not limited to, chromosome 3p and 9p [22-26]. Notably, 3p deletions are detected in almost 100% of SCLC and more than 90% of NSCLC cell lines [27]. Three *bona fide* lung cancer tumor suppressor genes, namely RBSP3 (AP20 region), NPRL2 and RASSF1A (LUCA region), were identified in the 3p21.3 region. In addition, the FHIT gene, of which inactivation is frequently found in various human cancers including lung cancer [28], is located at chromosome 3p14. Chromosome 9p21, which was reported to be most frequently deleted in lung cancer cell lines [29], contains two tumor suppressor genes, CDKN2A and CDKN2B.

The frequency of individual or combination of several MSIs or LOHs in lung cancer is typically lower than 70% [22,23,25]. However, this low prevalence may be increased by combining with other types of molecular markers, for example, methylation [26] and sputum cytology [24].

### 2.3. Methylation

DNA methylation is a common epigenetic mechanism that regulates gene expression. It involves the addition of a methyl group to the 5^th^ position of the cytosine base located 5′ to a guanosine in a CpG dinucleotide [30]. A CpG island, a contiguous stretch of DNA of at least 200 bp up to 4 kb, contains high CpG contents [31] and is often found in the promoter regions. Promoter hypermethylation is accompanied by histone modifications such as acetylation, methylation or phosphorylation of histone tails within the island [32]. This series of epigenetic alterations leads to a conformational change of the chromatin in the promoter regions, which prevents the RNA polymerase and other transcriptional regulatory proteins from accessing this region [32]. As a consequence, transcription is blocked and the gene becomes silenced. About 50% of human gene promoters contain CpG islands, which are generally unmethylated in the normal genome. However, in cancer, it is well established that CpG hypermethylation in the promoter region of genes that function in various pathways (such as DNA repair, cell cycle regulation, apoptosis and RAS signaling) is tightly associated with tumorigenesis [31].

There are several genes that are frequently silenced by promoter hypermethylation in lung cancer. *P16 (CDKN2A)* is a tumor suppressor gene that is hypermethylated at a prevalence of up to 67% in adenocarcinoma and 70% in squamous cell carcinoma of the lung [33]. It regulates the cell cycle and prevents excessive cell growth by regulating the tumor suppressor gene *RB1* and its protein, pRb. pRb prevents the cell from replicating damaged DNA by inhibiting its progression along the cell cycle through G1 into S. Intriguingly, inactivation of P16 has been proposed as an early step to immortalization [34,35], supporting its potential to be a useful biomarker for the early detection of cancer.

*MGMT* (*O*^6^-Methylguanine DNA methyltransferase) is a DNA repair enzyme that is inactivated in 24–48% of lung adenocarcinomas [33]. It antagonizes the genotoxic effects of alkylating agents. *MGMT* rapidly reverses alkylation, including methylation, at the *O*^6^ position of guanine by transferring the alkyl group to the active site of the enzyme [36]. Inactivation of *MGMT* in the cell allows the accumulation of O^6^-alkylguanine adducts in the DNA and can lead to transition mutations in genes such as *KRAS* and *TP53* [33].

*RASSF1A* (Ras-association domain family member 1A) is a tumor suppressor that is silenced in 40–60% and 100% of NSCLC and SCLC, respectively [33]. *RASSF1A* lacks apparent enzymatic activity but contains a Ras association (RA) domain and is potentially an effector of the Ras oncoprotein. *RASSF1A* modulates multiple apoptotic and cell cycle checkpoint pathways. Current evidence supports the hypothesis that it serves as a scaffold for the assembly of multiple tumor suppressor complexes and may relay pro-apoptotic signaling byK-Ras [37].

*DAPK* (death-associated protein kinase) is a pro-apoptotic, calcium/calmodulin-regulated serine/threonine protein kinase that is hypermethylated in both adenocarcinoma and squamous cell carcinoma at prevalences ranging from 30-48% [33]. DAPK mediates interferon-γ-mediated apoptosis and participates in a number of additional apoptosis-inducing pathways downstream of CD95 (Fas), tumor necrosis factor-alpha (TNF-α) and transforming growth factor-beta (TGF-β) [38]. Additionally, DAPK inactivation reduces the induction of p19^ARF^/p53, thus inactivating the p53-dependent pathway for apoptosis, suggesting that attenuation of p53 by loss of DAPK may be an important factor in transformation *in vivo* [39].

These four genes along with a few other genes, including *PAX5* and *GATA*, have been the major focus for examining the sensitivity/specificity of promoter methylation as sputum-based biomarkers for lung cancer detection. Among these, *P16* is reported to be the most frequently hypermethylated gene in sputum samples at prevalence ranging from 25–74% [40-42]. The combination of promoter methylation of more than one gene and/or with other types of biomarkers such as gene mutations or cytology seems to increase the sensitivity and specificity for cancer detection [40,41,43]. More importantly, hypermethylation of genes, particularly *P16*, may also be useful as biomarker for the prediction of the development of lung cancer among healthy high-risk smokers. Several studies have reported that hypermethylation in a panel of genes can be found in cancer-free smokers and is associated with increased risk of lung cancer [34,44-47]. The earlier occurrence of epigenetic abnormality than genetic modification such as gene mutation detected in histologically normal cells suggests that hypermethylation may be an early event during tumorigenesis. This may also explain the higher frequency of p16 hypermethylation than mutations in the *TP53* gene and/or the *KRAS* gene in sputum (see below) [47,48].

Gene methylation can be detected by methylation-specific PCR (MSP) [49] using sodium bisulfite, which converts all unmethylated, but not methylated, cytosines to uracil. Thus, bisulfite treatment introduces specific changes in the DNA sequence, which depends on the methylation status of individual cytosine residues. In the subsequent PCR reaction, primer pairs are designed to be “methylation-specific” by including sequences complementing only unconverted 5-methylcytosines. Recently, a modified version of MSP has been reported to increase the sensitivity. It involves a two-step nested PCR, which can improve the sensitivity by >50-fold compared to the conventional MSP [46].

One advantage of promoter hypermethylation as a biomarker compared to other genetic biomarkers (e.g., gene mutations, microsatellite abnormalities) is its reversibility by demethylating agent. Clinical trials for the treatment of myeloid neoplasms demonstrated that demethylating agents in combination with histone deacetylation inhibitor could demethylate certain genes in responding patients [50]. Thus, the reversibility of promoter hypermethylation in sputum biomarkers can be a powerful monitoring tool for evaluating the efficacy of demethylation therapy for lung cancer patients.

### 2.4. Mutation

There has been significant progress in the understanding of the genetic basis underlying lung cancer. Accumulation of mutations in genes that are involved in cell differentiation and growth leads to lung tumorigenesis [51]. Naturally, genetic mutations have been the area of focus for biomarker discovery.

*TP53* and *KRAS* are two of the most frequently mutated genes that are found in lung tumors and believed to be important in lung cancer pathogenesis. Thus, they are most well-studied genetic markers for lung cancer. *TP53* is a tumor suppressor and “guardian of the genome” [52]. Hence, mutations in the *TP53* gene are among the commonest in most cancers, including lung cancer [53]. A recent study found that about 65% of lung adenocarcinomas harbor mutations in *TP53*, which identifies *TP53* as the most frequently mutated gene in lung cancer [54]. *TP53* functions as an emergency brake by regulating tumor-preventing apoptosis and cell cycle progression. It modulates the transcription of genes that govern the major defense against tumor growth, which includes cell cycle arrest, apoptosis, maintenance of genetic integrity, inhibition of angiogenesis and cellular senescence [55]. Most mutations are missense and single-base substitutions distributed throughout the core DNA binding domain from exon 5 to exon 8, which abolish its function as a transcription factor [56].

*KRAS* is an oncogene that belongs to the Ras family. *RAS* genes encode a family of membrane-bound guanosine triphosphate (GTP)-binding proteins that regulate cell growth, differentiation, and apoptosis by interacting with multiple effectors, including those in the mitogen-activated protein kinase (MAPK), signal transducer and activator of transcription (STAT), and phosphoinositide 3-kinase (PI3K) pathways [57]. RAS proteins acquire transforming potential when an amino acid at position 12, 13, or 61 is replaced as a result of a point mutation in the gene [58]. These activating mutations result in the production of RAS protein that has impaired GTPase activity, leading to constitutive activation of RAS. *KRAS* mutations are frequently found in human malignancies and notably up to 60% in lung adenocarcinoma [54], being the second most frequently mutated gene in lung cancer. The fact that mutations in the *KRAS* gene occur almost exclusively at two hot spots (codons 12 and 13) facilitates the assessment of the presence of mutations at these codons by mutant allele enrichment method (See below).

Epidermal growth factor receptor (*EGFR*) belongs to a family of receptor tyrosine kinases (TKs) that transduce important growth factors signaling from the extracellular milieu to the cell. Structurally, each receptor is composed of an extracellular ligand binding domain, a transmembrane domain and an intracellular domain [59]. The receptor, which exists as an inactive monomer, undergoes conformational change upon the binding of a ligand such as EGF and forms a homo- or heterodimer. This event is then followed by autophosphorylation of the key tyrosine residues in the catalytic domain. Subsequently, proteins involved in downstream signaling events that control multiple cellular processes, including proliferation and survival, get recruited to the activated receptor via adaptor molecules [60]. Intracellular signaling is mediated mainly through the RAS-RAF-MEK-MAPK pathway, the PI3K-PTEN-AKT pathway, and the signal transducer and activator of transcription (STAT) pathway [61]. Two independent studies first reported the existence of somatic mutations in the TK domain of EGFR; the mutations are characterized by short deletions in exon 19 and point mutations (G719S, L858R, and L861Q) in exons 19 and 21 [62,63]. The mutations have been classified into three types. Class I mutations include short in-frame deletions that result in the loss of four to six amino acids (E746 to S752) encoded by exon 19. Class II mutations are single-nucleotide substitutions that may occur throughout exons 18 to 21. Class III mutations are in-frame duplications and/or insertions that occur mostly in exon 20 [64]. These mutations destabilize the kinase domain conformation and lead to constitutive activation, followed by uncontrolled activation of downstream signaling pathways including proliferation and survival.

The occurrence of mutations in EGFR and KRAS, which encodes a GTPase downstream of EGFR (see above), is mutually exclusive in lung cancers and they exhibit many contrasting characteristics such as clinical background, pathological features of patients harboring each mutation, and prognostic or predictive implications. Clinico-pathological features that correlate with *EGFR*-activating mutations include East Asian ethnicity, adenocarcinoma histology, female gender, and a history of never having smoked [64], while *KRAS* mutations are present in those with significant tobacco exposure. Lung cancers harboring the *EGFR* mutations are remarkably sensitive to *EGFR* tyrosine kinase inhibitors such as gefitinib or erlotinib, which has been a very important discovery impacting the clinical treatment of lung cancer. Because it is tightly associated with never-smokers, *EGFR* mutations are common targets for biomarker discovery for lung cancers in never-smokers but not in smokers.

Considering the great heterogeneity of cell types and low number of tumor cells found in sputum, it is crucial to employ a sensitive detection method that will distinguish the true positive signal of tumor cells from the vast majority of contaminating normal cells. Traditionally, to detect *KRAS* mutation at the 12^th^ and 13^th^ codons, the PCR-mutant allele enrichment (PCR-MAE) method was utilized, which has been reported to improve the sensitivity [65] compared to conventional PCR. However, this method is only suitable for the detection of specific known mutations because it requires the use of PCR primers that are complementary to the mutated sequence. For the detection of multiple and unknown mutations in *TP53*, the PCR-single stranded conformation polymorphism (PCR-SSCP) method is commonly used. Although this method may increase the sensitivity compared to conventional PCR, optimal conditions to sensitively separate alleles harboring various mutations have to be determined empirically, which can be tricky and time-consuming. As a result, studies employing similar methods had reported mutation frequencies ranging from lower than 10% frequency [25,48] to about 50% [65,66].

Notably, the rapid development of technology such as high-throughput sequencing now allows the detection of extremely rare mutant alleles in a highly heterogeneous sample. High-throughput sequencing is a method that parallelizes the sequencing process, producing thousands or millions of sequences at once. Choi *et al.* successfully identified two novel mutations in *EML4-ALK* gene in the sputum sample from a lung cancer patient using deep sequencing [67]. The high-throughput nature of this system will also expand the capacity of screening, facilitating the examination of mutations in multiple genes simultaneously. Recently genome-wide screening studies to identify frequently mutated genes in lung cancer (e.g., *STK11*, *LRP1B*, *LPHN3*) have provided an exciting new insight into the discovery of potentially novel gene candidates for lung cancer in addition to *TP53* and *KRAS* [54,68]. The findings of these studies are promising because they offer additional and perhaps better target (or combination of targets) for the early detection of lung cancer. Moreover, identification of the functions of these candidate genes and the consequences of mutations during the early stage of tumorigenesis will provide rationale for the use of mutations of these candidate genes for the early detection of cancer.

In summary, genes that harbor limited number of hot spot mutations (e.g., *KRAS* mutations exclusively occur on codon 12 or 13) will be more practical for large-scale screening than genes (e.g., *TP53*) with numerous mutations scattered throughout the gene, which will complicate the screening strategy. In addition, since *TP53* mutations are found in almost every type of human cancer, an ideal biomarker for lung cancer should be a gene with mutation that is specifically associated with lung cancer. Lastly, the use of high-throughput sequencing for evaluating mutations in these new candidate genes in addition to *TP53* and *KRAS* in sputum will make gene mutation screening a powerful tool for lung cancer detection.

### 2.5. microRNAs

MicroRNAs (miRNAs) are a class of short (∼22 nucleotides long) and highly conserved non-coding RNAs involved in numerous developmental processes. They regulate gene expression by incomplete base-pairing to a complementary sequence in the 3′ untranslated region of a target mRNA, which leads to mRNA degradation or translation repression [69]. Deregulation of miRNAs has been linked to cancer initiation and progression, indicating that miRNAs may act as tumor suppressor genes or oncogenes and can target apoptosis [70]. It is believed that miRNAs regulate signal transduction pathways and oncogenic pathways leading to lung cancer. Interestingly, several studies have identified differential miRNA expression profiles associated with lung cancers [71-74], which suggests that miRNA signatures are potential biomarkers for lung cancers.

Although miRNA has recently emerged as powerful molecular biomarkers for the detection of cancer, its potential as a sputum-based biomarker has not been fully explored. Jiang *et al.* recently reported two studies evaluating differential miRNA expression in sputum samples from lung cancer patients [74,75]. Although they demonstrated that miRNAs existed stably in sputum and could be amplified robustly [76], the studies are somewhat limited because the candidate miRNA molecules were initially discovered from tissue, not sputum, followed by validation of the selected candidates in sputum. Although this strategy can still provide valuable information, screening the entire miRNA transcriptome in sputum would be more desirable for the discovery of novel sputum-based miRNA biomarkers. However, one of the practical hindrances to the latter is that the normalization methods for miRNA, especially in an extremely heterogeneous sample like sputum, have not been fully established yet, which makes it challenging to assess the validity of miRNA profiling data. In addition, due to the inevitable variability in the sputum cell composition among samples, which is a major confounding factor, interpreting any differential miRNA expression can be challenging. Therefore, although sputum-based miRNAs have great potential as biomarkers for lung cancer, a vigorous system for evaluating the differential miRNA expression in sputum has to be developed.

## 3. Conclusions and Future Directions

Although biomarker discovery in sputum has been promising, the clinical applications have been limited, mainly because of the inconsistent results and low sensitivity. One key to tackling these issues is obtaining more bronchial epithelial cells (BECs) in sputum, which is typically less than 5% [77,78]. Increasing the number of BECs will eventually result in more cancer cells in sputum. One way to achieve this might be using sputum induction instead of spontaneous collection. However, the effect of different collection methods on the resultant percentage of bronchial epithelial cells in sputum has not been actively discussed in the literature. In addition, published studies frequently omit the detailed description of collection methods for sputum, which makes it difficult to deduce the effect collection methods on cell populations from these studies. Therefore, careful assessment of the sputum cell composition collected by different methods is urgently required. More importantly, standardized sputum collection and processing protocols should be established to minimize the inconsistencies resulting from different laboratories using different methods.

Enriching the bronchial epithelial cells after collection is another strategy to increase the percentage of bronchial epithelial cells and get rid of the potentially confounding cell types such as macrophages and neutrophils. A few different enrichment methods have been proposed, including laser microdissection and magnetic-assisted cell sorting (MACS). When epithelial cells in lung cancer sputum samples were selectively captured by microdissection, the sensitivity for the detection of mutations in *TP53* and *KRAS* increased about three times (∼46%) compared to using the entire sputum cells [79]. Furthermore, the same research group was able to detect mutations in *TP53* (14.1%) and *KRAS* (1.1%) in lung cancer-free, healthy high-risk group using laser microdissection [80]. MACS is another common cell selection method. Typically, bronchial epithelial cells are negatively selected by using anti-CD14 and anti-CD16 magnetic beads to deplete the macrophages and the neutrophils, respectively, which together account for the majority of the cell populations in sputa. It has been reported that bronchial epithelial cells can be enriched from 1.1% (unsorted) up to 40% after MACS, producing an average of 43% purity in the processed samples and resulting in an average 36-fold enrichment [81]. These studies suggest that by selectively enriching the bronchial epithelial cells, molecular abnormalities in sputum samples could be detected with higher sensitivity.

In spite of the efforts to enrich for BECs in sputum, the rare presence of cancer cells in sputum strongly argues for biomarkers that are present in non-cancerous epithelial cells in early stage cancer or in high-risk smokers. Thus, examining molecular abnormalities, which have been identified in tumor tissue, in sputum samples may not be very successful. This requires the discovery of novel sputum-based biomarkers that are caused by lung cancer risk factors, namely tobacco smoking, and are harbored in pre-malignant cells. An exciting example is a recent study by the Backman group, which described abnormal nanostructure architecture in microscopically normal-appearing buccal epithelial cells from lung cancer patients using a novel optical technology, partial wave spectroscopic microscopy, which is exquisitely sensitive to the nanoarchitectural manifestation of the genetic/epigenetic alterations of field carcinogenesis [82]. This suggests the possibility that the histologically normal airway epithelium harbors potentially powerful biomarkers that await discovery. Thus, the combination of epithelium cell enrichment, more sensitive detection methods and novel field cancerization biomarkers will lead to the discovery of more accurate and reliable sputum-based biomarkers that can be used for the early detection of lung cancer.
